# *Burkholderia pseudomallei* in Environment of Adolescent Siblings with Melioidosis, Kerala, India, 2019

**DOI:** 10.3201/eid2806.211526

**Published:** 2022-06

**Authors:** Praveena Bhaskaran, Vinitha Prasad, Anusha Gopinathan, Tushar Shaw, Suchitra Sivadas, Chandrasekhar Jayakumar, Soumi Chowdhury, Aparna Dravid, Chiranjay Mukhopadhyay, Anil Kumar

**Affiliations:** Amrita Institute of Medical Sciences and Research Centre, Amrita Vishwa Vidyapeetham, Kochi, India (P. Bhaskaran, V. Prasad, S. Sivadas, C. Jayakumar, A. Kumar);; SRM Medical College Hospital and Research Centre, Chennai, India (A. Gopinathan);; Ramaiah University of Applied Sciences, Bangalore, India (T. Shaw);; Manipal Academy of Higher Education, Manipal, India (T. Shaw, S. Chowdhury, A. Dravid, C. Mukhopadhyay)

**Keywords:** Melioidosis, Burkholderia pseudomallei, environmental surveillance, lymphadenitis, sepsis, bacteria, Kerala, India

## Abstract

In 2019, *Burkholderia pseudomallei* was isolated from the backyard of 2 siblings with melioidosis in Kerala, India. This finding highlights the value of healthcare providers being aware of risk for melioidosis in febrile patients, of residents taking precautions when outside, and of increasing environmental surveillance for *B. pseudomallei* in this region.

Melioidosis is a potentially life-threatening infection caused by the soil-dwelling bacteria *Burkholderia pseudomallei.* The clinical spectrum of disease ranges from mild localized lesions to fulminant sepsis (*1*,*2*). The disease is endemic to Southeast Asia and Australia. Recently, the southern part of India has emerged as a melioidosis hot spot; infection in children accounts for 8% of infections (*2*,*3*). The risk of children acquiring infection is high because of the likelihood of environmental exposure either through wounds acquired while playing in contaminated muddy water or by inhalation of aerosols containing the bacteria during the rainy season (*4*–*6*). We report melioidosis in 2 siblings from Kerala, India, and the subsequent isolation of *B. pseudomallei* from the soil in their backyard.

## The Study

**Figure 1:**
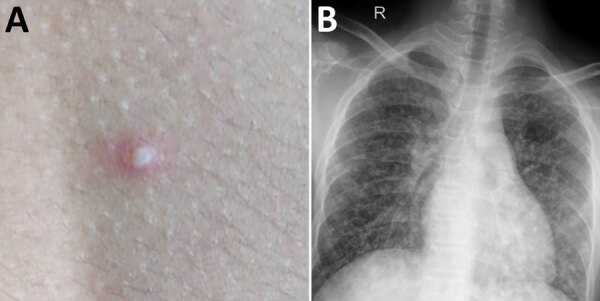
Images of 15-year-old boy (patient 1) with melioidosis, Kerala, India, 2019. A) Vesicular lesion on trunk. B) Chest radiograph showing diffuse heterogeneous opacities suggestive of acute respiratory distress syndrome.

In July 2019, at the onset of the monsoon season, a 15-year-old boy from southern India (patient 1) was examined because of fever for 1 week along with cough, myalgia, headache, vomiting, and diarrhea, followed by rashes over the trunk and breathlessness for 1 day. At hospital admission, the child was febrile, tachycardic, and tachypneic; his blood pressure was 85/67 mm Hg, and oxygen saturation on room air was 90%. He had a few discrete pustular lesions over the trunk (Figure 1, panel A). Chest examination revealed bilateral crackles. Initial laboratory studies showed leukocytosis; increased levels of inflammatory markers; and abnormal liver function, renal function, and coagulation profile, suggestive of multiorgan dysfunction (Table). The patient was admitted to the intensive care unit, and noninvasive ventilation and inotropic support were initiated along with empiric intravenous (IV) administration of piperacillin/tazobactam. Acute respiratory distress syndrome (Figure 1, panel B) with respiratory failure subsequently developed, and the patient was emergently intubated. Antimicrobial drugs were changed to meropenem and vancomycin. Despite these measures, the patient deteriorated rapidly, respiratory status and shock worsened, and he died within 24 hours after hospital admission. 

**Table:** Clinical and laboratory characteristics of 2 siblings with melioidosis, Kerala, India, 2019*

Characteristic	Patient 1, 15-year-old boy†	Patient 2, 12-year-old boy‡
Clinical		
Comorbidities	None	None
Date of admission	Jul 1	Aug 8
Clinical condition	Pneumonia, ARDS, septic shock	Fever, sore throat, cervical lymphadenopathy
Laboratory		
Hemoglobin (ref 12.7–17.7), g/dL	13	13.4
Total leukocytes (ref 5,500–15,500), cells/μL	3,190	14,260
Neutrophils (ref 55–70), %	90	73.1
Thrombocytes (ref 150–400), K/µL	100	223
AST (ref 0–51), IU/L	139.4	21.2
ALT (ref 0–39), IU/L	98	16.3
Creatinine (ref 0.4–1.06), mg/dL	26.4/1.13	0.54
BUN (ref 5–20), mg/dl	26.4	14.3
Ferritin (ref 36–311), ng/mL	5,448	1374
Procalcitonin (refR <0.5), ng/mL	6.8	0.36
C-reactive protein (ref <10), mg/L)	319	16.86
Culture (source)	*Burkholderia pseudomallei* (blood, skin lesions)	*B. pseudomallei* (lymph node, throat swab)
Blood PCR	Positive for *Burkholderia mallei*/B. *pseudomallei*	Not detected
Diagnosis (manifestations)	Melioidosis (pneumonia, ARDS, sepsis)	Melioidosis (lymphadenitis)
Treatment	Piperacillin/tazobactam, meropenem, vancomycin	Ceftazidime, meropenem, trimethoprim/sulfamethoxazole
Outcome	Died Jul 2	Discharged Sep 20

*ARDS, acute respiratory distress syndrome; ALT, alanine aminotransferase; AST, aspartate aminotransferase; ref, reference range.

Multiplex real-time PCR (FastTrack Diagnostics, https://www.imogena.pl) of a blood sample for common tropical diseases was positive for *Burkholderia mallei*/*pseudomallei*. Cultures of blood and of pus aspirated from the skin lesions yielded nonfermentative gram-negative bacilli, which the Vitek 2 system (bioMérieux, https://www.biomerieux.com) identified as *B. pseudomallei*. Isolate identity was confirmed at the Centre for Emerging and Tropical Diseases, Manipal Academy of Higher Education (Manipal, India), by monoclonal antibody–based latex agglutination test and PCR specific for the *B. pseudomallei*–specific *TTSS1* gene (*7*,*8*). Both isolates were sensitive to amoxicillin/clavulanic acid, ceftazidime, cotrimoxazole, doxycycline, and meropenem.

One month later, the patient’s 12-year-old brother (patient 2) was examined for fever of 3 weeks’ duration along with neck swelling and sore throat of 2 weeks’ duration. He received amoxicillin/clavulanate with no response. His vital signs were stable. Throat examination revealed bilateral tonsillar exudates. His left posterior cervical lymph node was 3 × 2 cm, firm, and nontender. His initial laboratory parameters were unremarkable (Table). Histopathology of the cervical node showed necrotizing lymphadenitis. Culture of lymph node tissue and throat swab grew *B. pseudomallei* with sensitivity identical to that of the isolate from his sibling. IV ceftazidime was administered, but because fever persisted, IV trimethoprim/sulfamethoxazole was added on day 5. Whole-body fluorodeoxyglucose positron emission tomography magnetic resonance imaging did not show any deep-seated abscesses. Because the fever continued to persist, ceftazidime was changed to meropenem by day 10. Fever subsided by day 14, and IV meropenem and trimethoprim/sulfamethoxazole were continued for 2 more weeks. The patient was discharged with instructions to take oral trimethoprim/sulfamethoxazole for 4 months. As of 30 months later, the child remained healthy. 

**Figure 2:**
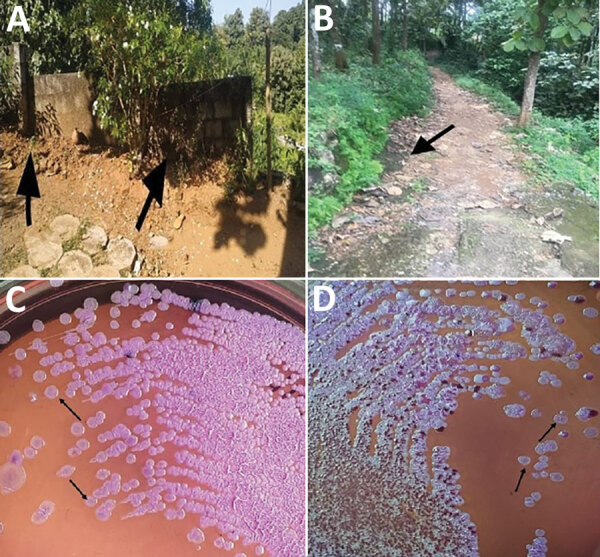
Cultures from environment of siblings with melioidosis, Kerala, India, 2019. A, B) Sites of soil collection (arrows) near patients’ house. C, D) Ashdown agar culture showing growth of pink wrinkled colonies with metallic sheen (arrows) after 72-h incubation, typical of *Burkholderia pseudomallei*.

Melioidosis cases among siblings within a span of 1 month suggested exposure to a common environmental source. In November 2019, we sampled the patients’ environment and collected 20 soil samples from an area of 1,000 square feet around the patients’ house (Figure 2). We collected and processed soil dug from the flower bed and soil at 30 cm deep according to published consensus guideline for detection of *B*. *pseudomallei* in soil (*9*). We tested samples by culture and PCR for the presence of the bacteria. Of the 20 samples, 3 were positive for *B. pseudomallei* by culture (Figure 2) and 18 showed positive signal in the qualitative PCR targeting the *TTSS1* gene for *B. pseudomallei*. Antimicrobial susceptibility was the same for all 3 environmental isolates as for the clinical strains. Multilocus sequence typing of the clinical and environmental isolates indicated that the sequence type (ST) of the strain from the 12-year-old sibling matched that of the environmental isolates (ST 1944). The sequence type of the isolate from the 15-year-old sibling was different (ST 1556). Interview of the family indicated that both siblings had spent time playing outside the house during the rain, a known risk factor for acquiring melioidosis. The other family members were made aware of the risk factors of acquiring the disease from their environment and were advised to take preventive measures. They were asked to inform their doctor regarding the possibility of melioidosis when seeking medical care for any febrile illness or respiratory infection. However, they have remained healthy throughout the follow-up period.

## Conclusion

Melioidosis is an underdiagnosed disease in India because of its diverse clinical manifestations, clinical overlap with other conditions such as tuberculosis, limited awareness by physicians and the general public, and laboratory constraints (*1*–*3*). Previous studies have reported cases from eastern, northeastern, and southern India (*3*,*10*). Both patients described in this report became ill during the monsoon season, when it rains heavily in Kerala. A recent study from southern India indicated that 90% of the children became ill during the monsoon season (*5*). Studies have shown a correlation between the disease and rainfall intensity (*3*,*11*). 

A recent study from India reported frequent respiratory involvement and bacteremia in adults with acute cases (*10*). A study conducted in Southeast Asia reported that localized manifestations were more common in children; 46%–63% had either cutaneous lesions or lymphadenopathy (*12*). A similar study from India reported that among total cases of melioidosis, 8% were in children and the mortality rate was 9%. In that study, 54% of children had disseminated illness and 46% had localized lesions (*5*). 

Fatal melioidosis in siblings and isolation of *B*. *pseudomallei* from their environment have been recently reported from Mexico (*13*). Cases of *B. pseudomallei* infection among siblings with cystic fibrosis have been reported from Australia (*14*). *B. pseudomallei* was also isolated from 5 of the 40 soil and water samples collected from the coastal region of Malabar, Kerala, during the 2016 rainy season (*15*). We report simultaneous isolation of *B. pseudomallei* from humans and from their immediate surrounding environment.

This study highlights the perpetual risk faced by family members of affected persons and other persons residing in a region where cases of melioidosis have been reported. It also highlights the value of seeking appropriate medical care at the onset of any febrile illness, because early diagnosis and treatment can decrease illness and death associated with the disease. Public health officials should emphasize additional precautions in melioidosis-endemic regions (e.g., not going out in the field during heavy rainfall, not walking barefoot, and not drinking water from natural reservoirs in melioidosis-endemic areas). Healthcare workers and the community should be aware of the risk for melioidosis, especially during the rainy season, and diagnostic testing should be strengthened to increase prompt identification and treatment. Furthermore, the National Centre for Disease Control should add melioidosis to the notifiable disease list in India to enable increased environmental surveillance and provide data for melioidosis risk mapping.

## References

[1] Wiersinga WJ, Currie BJ, Peacock SJ. Melioidosis. N Engl J Med. 2012;367:1035–44. 10.1056/NEJMra1204699 22970946

[2] Wiersinga WJ, Virk HS, Torres AG, Currie BJ, Peacock SJ, Dance DAB, et al. Melioidosis. Nat Rev Dis Primers. 2018;4:17107. 10.1038/nrdp.2017.107 29388572 PMC6456913

[3] Mukhopadhyay C, Shaw T, Varghese GM, Dance DAB. Melioidosis in South Asia (India, Nepal, Pakistan, Bhutan and Afghanistan). Trop Med Infect Dis. 2018;3:51. 10.3390/tropicalmed3020051 30274447 PMC6073985

[4] Sanderson C, Currie BJ. Melioidosis: a pediatric disease. Pediatr Infect Dis J. 2014;33:770–1. 10.1097/INF.0000000000000358 24732448

[5] Mukhopadhyay C, Eshwara VK, Kini P, Bhat V. Pediatric melioidosis in Southern India. Indian Pediatr. 2015;52:711–2. 26388638

[6] Pagnarith Y, Kumar V, Thaipadungpanit J, Wuthiekanun V, Amornchai P, Sin L, et al. Emergence of pediatric melioidosis in Siem Reap, Cambodia. Am J Trop Med Hyg. 2010;82:1106–12. 10.4269/ajtmh.2010.10-0030 20519608 PMC2877419

[7] Duval BD, Elrod MG, Gee JE, Chantratita N, Tandhavanant S, Limmathurotsakul D, et al. Evaluation of a latex agglutination assay for the identification of *Burkholderia pseudomallei* and *Burkholderia mallei.* Am J Trop Med Hyg. 2014;90:1043–6. 10.4269/ajtmh.14-0025 24710616 PMC4047727

[8] Novak RT, Glass MB, Gee JE, Gal D, Mayo MJ, Currie BJ, et al. Development and evaluation of a real-time PCR assay targeting the type III secretion system of *Burkholderia pseudomallei.* J Clin Microbiol. 2006;44:85–90. 10.1128/JCM.44.1.85-90.2006 16390953 PMC1351940

[9] Limmathurotsakul D, Dance DAB, Wuthiekanun V, Kaestli M, Mayo M, Warner J, et al. Systematic review and consensus guidelines for environmental sampling of *Burkholderia pseudomallei.* PLoS Negl Trop Dis. 2013;7:e2105. 10.1371/journal.pntd.0002105 23556010 PMC3605150

[10] Koshy M, Jagannati M, Ralph R, Victor P, David T, Sathyendra S, et al. Clinical manifestations, antimicrobial drug susceptibility patterns, and outcomes in melioidosis cases, India. Emerg Infect Dis. 2019;25:316–20. 10.3201/eid2502.170745 30666953 PMC6346473

[11] Currie BJ, Jacups SP. Intensity of rainfall and severity of melioidosis, Australia. Emerg Infect Dis. 2003;9:1538–42. 10.3201/eid0912.020750 14720392 PMC3034332

[12] Foong YW, Tan NW, Chong CY, Thoon KC, Tee NW, Koh MJ. Melioidosis in children: a retrospective study. Int J Dermatol. 2015;54:929–38. 10.1111/ijd.12837 25771733

[13] Alvarez-Hernandez G, Cruz-Loustaunau D, Ibarra JA, Rascon-Alcantar A, Contreras-Soto J, Meza-Radilla G, et al. Description of two fatal cases of melioidosis in Mexican children with acute pneumonia: case report. BMC Infect Dis. 2021;21:204. 10.1186/s12879-021-05910-5 33622263 PMC7903701

[14] Holland DJ, Wesley A, Drinkovic D, Currie BJ. Cystic fibrosis and *Burkholderia pseudomallei* infection: an emerging problem? Clin Infect Dis. 2002;35:e138–40. 10.1086/344447 12471591

[15] Peddayelachagiri BV, Paul S, Nagaraj S, Gogoi M, Sripathy MH, Batra HV. Prevalence and identification of *Burkholderia pseudomallei* and near-neighbor species in the Malabar coastal region of India. PLoS Negl Trop Dis. 2016;10:e0004956. 10.1371/journal.pntd.0004956 27632353 PMC5025242

